# On the Submucous Division of the Sphincter in Affections of the Anus

**Published:** 1846-08

**Authors:** 


*On the Submucous Division of the Sphincter in Affections of the Anus.* By M. Demarquay.—The division of the sphincter, as practised by Boyer, frequently fails, and is not devoid of danger; but all its advantages may be obtained and inconveniences obviated by operating upon it beneath the mucous membrane. MM. Guerin, Velpeau, Blandin, and the author have employed this modification with great success, and it is the object of this paper to spread a wider knowledge of the proceeding by detailing several of the cases which have been treated by the two last.

Submucous myotomy is especially calculated for the relief of *spasmodic action and contraction of the sphincter ani.* It is often difficult to say where one of these affections ends and the other begins, for they are so intimately connected that the one may be said to be a consequence of the other: for if the same irritating causes which induce spasmodic contraction of the muscle continue in operation, this will then become permanent. When the contraction is well marked the anus seems deeper seated than ordinary, the borders of the external sphincter being seen projecting and the pleats of the region well-marked. If hæmorrhoids or mucous membrane has become engaged within the sphincter, the compression gives them a violet colour, and they may even become gangrenous. The finger is introduced with difficulty, and with pain to the patient, and is much constricted by the muscle.

Spasmodic contraction, requiring the operation, may manifest itself under various circumstances, but it is especially in its prevention of the *extraction of foreign bodies* from the rectum, that it comes under our notice. It may also manifest itself in two very important affections, viz., *prolapsus of the rectum* and the *sudden appearance of large hæmorrhoids.* In this last case, the constriction may be so considerable as to induce gangrene, or at all events prevent the return of the protruded parts.

Contraction of the sphincter plays an important part in three affections, viz., constipation, old hæmorrhoids, and fissure of the anus.

(1.) Prolonged and ostinate *constipation* not unfrequently gives rise to it. The indurated matters contained in the gut irritate it, and induce, first spasmodic and then more permanent, contraction; and authors have certainly sometimes mistaken the effect for the cause, in regarding the constipation as produeed by the contraction. In mentioning constipation as a cause of contraction, it is not intended to recommend combating it by a surgical operation. It is a complex condition of which this contraction only forms a portion.

(2.) When *hæmorrhoids* have long existed, and become frequently congested and inflamed, it is by no means rare to find contraction induced. In this case they are forced out at stool, but can only be partially returned. They inflame; defæcation becomes more and more difficult, and fissures and hæmorrhages result. Several examples are given in which this state of things are effectually relieved by the submucous section.

(3.) *Fissure of the Anus* is so intimately connected with contraction of the sphincter that Boyer regarded them as the same disease. But we have already seen contraction may exist without fissure, and the question is, when the two affections co-exist, which has preceded the other. In the great majority of cases a more or less obstinate constipation, a state often giving rise to contraction, has preceded the fissure. Authors, however, have stated that they have observed fissure without contraction, and if they are correct we may explain such cases by the varieties of the disease admitted by M. Blandin. He states that the fissure may be seated above, opposite, or below the sphincter. In the last case it may exist without contraction.

The author has analysed all the cases of fissure of the anus which have been published, as far as he knows, since the time of Boyer. They amount to 53, of several of which very insufficient particulars are given. Of these persons, 30 were men and 23 women, in opposition to Boyer's assertion that females are most liable to the disease. In six instances the fissure was double, triple, or quadruple. The age is only recorded in 42 cases, the disease being most common between 20 and 30, and 30 and 40. In 26 of the cases constipation, and in six cases hæmorrhoids, are described as existing.

Seven cases of fissure operated upon by M. Blandin are given, in all of which complete and immediate relief succeeded to the most intense suffering. Prior to commencing the operation the rectum should be well-cleared out, so as to obviate the necessity of going to stool shortly after it. An assistant must raise the side of the buttocks opposite to that on which the section is about to be made. This last is always practised at one of the sides of the anus, so that the sphincter may be divided through its middle. An ordinary *tenotome* may be used, or a bistoury which M. Blandin has contrived. The former is, however, not long enough, and its point is not sufficiently guarded, so that there is danger of penetrating the mucous membrane with it. M. Blandin's bistoury is protected by a moveable sheath, and he can either use it for puncture or incision, or as a flattened probe, accordingly as he wishes to divide parts, or merely to pass it between the muscle and mucous membrane. Whichever may be selected, the operation is simple. 1. Make a small opening in the skin. 2. Introduce the finger into the rectum, at the same time that the skin on each side of the anus is stretched. 3. Pass the tenotome between the mucous membrane and the sphincter. 4. Divide the latter. The puncture of the skin is most conveniently made at from 8 to 12 lines from the anus. The instrument must be passed in very gently so as to detach parts as little as possible. When the division has been made a kind of crack is heard, and the finger which is in the rectum feels the space between the two divided portions of the muscle. Although not absolutely necessary, the patient had better keep his bed for a few days after; and he should live very abstemiously, as it is desirable he should not go to stool for three or four days, after which there is no fear of rupture of the cicatrix. Occasionally the section of the sphincter on one side only is insufficient. The fissure does not after the section of the muscle require any special treatment.

*Ibid, from Archives Generales.*

[When the dreadful and prolonged suffering incident to fissure of the anus is considered, the substitution of this simple operation for that of Boyer's must be regarded as of one the most valuable applications of myotomy.—*Med. Chir. Rev.*]

